# Post-COVID-19 Thyroid Function Changes in Primary Care: A Retrospective Observational Study

**DOI:** 10.3390/healthcare14183013

**Published:** 2026-09-15

**Authors:** Dorota Szydlarska, Marta Zuzanna Ciechomska, Andrzej Śliwczyński, Alicja Jakubowska, Waldemar Wierzba, Olga Maguza

**Affiliations:** 1 Department of Family Medicine, National Medical Institute of the Ministry of the Interior and Administration, 02-507 Warsaw, Poland; dorota.szydlarska@pimmswia.gov.pl (D.S.); alicja.jakubowska@pimmswia.gov.pl (A.J.); waldemar.wierzba@pimmswia.gov.pl (W.W.); 2 Department of Family Medicine, Medical University of Warsaw, 02-091 Warsaw, Poland; 3 Faculty of Health Sciences, WSEI University, 20-209 Lublin, Poland; andrzej.sliwczynski@pimmswia.gov.pl; 4 Faculty of Medicine, Lazarski University, 02-662 Warsaw, Poland; 49239@lazarski.edu.pl

**Keywords:** COVID-19, post-COVID, thyroid function, primary care, subacute thyroiditis, TSH, retrospective study

## Abstract

**Highlights:**

**What are the main findings?**

- COVID-19 was associated with a significant reduction in the mean TSH level in primary care patients.
- The fT3 and fT4 concentrations remained unchanged, suggesting predominantly subclinical alterations.

**What are the implications of the main findings?**

- Thyroid function changes after COVID-19 were predominantly subclinical and may go unrecognized in primary care settings.
- Increased awareness may support the earlier identification of patients requiring further evaluation.

**Abstract:**

**Background:** Coronavirus disease 2019 (COVID-19) has been associated with thyroid dysfunction; however, evidence from primary care settings remains limited. Given that most patients with COVID-19 are managed outside hospital settings, understanding thyroid function changes in real-world primary care is clinically relevant. **Methods**: This retrospective study included 373 adult outpatients with confirmed COVID-19 and no prior thyroid disease, who were managed in a Family Medicine Clinic in Warsaw between January of 2022 and December of 2024. Thyroid function tests (yielding TSH, fT3, and fT4 levels) obtained within three months before and after infection were analyzed. Subacute thyroiditis (SAT) was assessed in symptomatic patients via thyroid ultrasound. Paired comparisons were performed, with *p* < 0.05 considered statistically significant. **Results**: A significant reduction in the mean TSH level was observed following COVID-19 compared with pre-infection values (mean 2.39 vs. 2.17 mIU/L; *p* < 0.05), while fT3 (2.93 vs. 2.98 pg/mL) and fT4 (1.31 vs. 1.30 ng/dL) concentrations remained unchanged. No significant associations were found between thyroid function changes and age or sex. **Conclusions**: In this real-world primary care population, lower TSH concentrations were observed following COVID-19 while peripheral thyroid hormone levels remained unchanged, suggesting predominantly modest changes in thyroid function parameters. Given the retrospective study design and the absence of a control group, these findings should be interpreted with caution. Further prospective studies are needed to clarify the clinical significance of post-COVID-19 thyroid function changes.

## 1. Introduction

The COVID-19 pandemic, caused by the severe acute respiratory syndrome coronavirus-2 (SARS-CoV-2), has led to a wide range of complications beyond the acute phase of infection. In this context, one of the most important problems is the activation of various diseases—especially autoimmune and inflammatory conditions—following recovery from COVID-19. This phenomenon, which has been observed in both adults and children, highlights the complexity of interactions between the virus and the human immune system. Inflammatory and autoimmune conditions in patients who have recovered from COVID-19 may be triggered as a result of several different mechanisms [1]; the infection has been observed to trigger both innate and adaptive immune responses, in some cases resulting in the production of autoantibodies. These antibodies can attack various tissues and organs, resulting in inflammation that is experienced by patients in various ways, including as pain in specific locations (e.g., rheumatoid arthritis (RA); systemic lupus erythematosus; inflammation of tissues and vessels; inflammation within the visual system; and ear, nose, and throat (ENT) diseases) [2,3,4,5]. The mechanisms potentially contributing to the activation of inflammation can be analyzed according to criteria such as molecular mimicry, comorbidity activation, and likely loss of immune tolerance [6,7]. Loss of immune tolerance may result from SARS-CoV-2-induced immune dysregulation, promoting autoreactive lymphocyte activation and autoantibody production, which may contribute to autoimmune thyroid disease development. Post-COVID-19 activation of rheumatic diseases [8,9,10,11]; tissue and vessel inflammation [10,12]; neurological disorders [13]; and endocrine and metabolic disorders have been reported [4,12,14]. The first place that patients experiencing unusual symptoms (including pain in unexpected places) report to is primary health care (PHC) centers. Primary health care centers in Poland provide care to patients who have declared a physician affiliated with a particular center to be their primary care physician. This positioning of PHC within the health care system determines the nature of health problems for which patients visit PHC centers. In a very general way, PHC patients can be divided into two groups: namely, those with chronic health problems (usually requiring continuation of therapy prescribed by a specialist) and those with emergency health problems. Pain-related complaints are among the most common reasons for primary health care center visits, and their management usually begins with symptomatic treatment.

Subacute thyroiditis (SAT)—also known as subacute granulomatous thyroiditis or De Quervain’s thyroiditis—is a disease characterized by sudden-onset neck pain and thyrotoxicosis symptoms (predominantly palpitations) of varying severity. The most common symptom of SAT is anterior neck pain radiating to the jaw and the ear, although pain-free cases have also been reported [15,16,17,18,19]. A frequently reported complaint is fever, frequently exceeding 39 °C and increasing especially at night, possibly accompanied by cardiac palpitations; in addition, patients often complain of fatigue and malaise. In clinical practice, the presentation of SAT is dominated by symptoms that are identical to and consistent with those of upper respiratory tract infections, and distinguishing between the two in the early phase can be very difficult in primary care contexts. A key issue is the selection of patients to undergo detailed endocrinological diagnostic procedures following mild COVID-19 that does not require hospitalization. Laboratory tests, as carried out in the course of SAT, can reveal features of hyperthyroidism as well as elevated erythrocyte sedimentation rate (ESR) and increased C-reactive protein (CRP) serum levels, which are typically observed in upper respiratory tract infections. In SAT patients, ultrasound examination of the thyroid gland typically reveals hypoechoic areas with indistinct borders and reduced vascularization. Although the cause of SAT has not yet been established and the mechanisms responsible for the triggering of SAT in post-COVID-19 patients have not been examined in detail, the following causes are assumed to underlie the development of the disease (as well as other diseases that can be observed in such patients):

1. Molecular mimicry—the SARS-CoV-2 spike protein may present structural similarities to thyroid antigens, thus triggering an autoimmune response [20,21];
2. Immune–inflammatory response—vaccines can induce an exaggerated immune response, leading to thyroiditis [2,3];
3. Adjuvant-induced autoimmunity—adjuvants in vaccines may contribute to autoimmune/inflammatory syndrome induced by adjuvants (ASIA syndrome) [2,3].

These mechanisms are supported by the observation that SAT can develop regardless of the type of vaccine, suggesting the viral proteins rather than the vaccine platform as the potential trigger [22].

SAT is a disease with a confirmed genetic basis, while viral infection is believed to be the direct causative agent. Notably, the 2019 coronavirus disease (COVID-19) pandemic led to millions of confirmed cases worldwide. The severe acute respiratory syndrome coronavirus 2 (SARS-CoV-2) exhibits significant tissue tropism, resulting mainly in interstitial pneumonia and inducing a turbulent inflammatory response. In severe cases, the latter involves the cardiovascular, gastrointestinal, and nervous systems, and can lead to renal failure and coagulation disorders. Although no association between coronaviruses and the development of SAT had been described prior to the COVID-19 pandemic event [23], observations regarding the previous severe acute respiratory syndrome coronavirus 1 (SARS-CoV-1) indicated possible damage to thyrocytes with subsequent fibrosis, consistent with the presentation of subacute thyroiditis [24]. Furthermore, the development of thyroid disease in the course of severe acute respiratory syndrome (SARS) has been linked to various mechanisms of thyroid damage, including excessive immune response, infection-related immune deficiency, and direct cellular damage [25]. Angiotensin-converting enzyme type 2 (ACE2)—which is richly localized in thyroid cells and serves as the viral cell pathway receptor—plays an essential role in SARS-CoV-2 infection [26,27]. Due to the resulting increased affinity of SARS-CoV-2 for the thyroid gland, attention has been drawn to the potential risk of thyroid diseases developing in COVID-19 patients. While cases of Graves’ disease and Hashimoto thyroiditis have also been described [28], SAT remains the most common thyroiditis associated with COVID-19 [8,29].

The objective of this study was to evaluate changes in thyroid function in COVID-19 outpatients not requiring assisted ventilation or oxygen therapy.

## 2. Materials and Methods

The study group included 1210 adult patients with a history of COVID-19 who remained under the continuous care of the medical staff of the Family Medicine Outpatient Clinic of the National Medical Institute of the Ministry of the Interior and Administration Medicine in Warsaw between the beginning of January of 2022 and the end of December of 2024. Among the 1210 patients that were initially screened, patients with previously diagnosed thyroid disease, a history of thyroidectomy, or missing thyroid function test results within the predefined study window were excluded. Consequently, 373 patients met all the inclusion criteria and were included in the final analysis. Women made up the majority of the study population (*n* = 267). The average age was 63.8 ± 16.7 years, with a median age of 66 years. Thyroid hormone laboratory assays, including measurements of thyroid-stimulating hormone (TSH), free triiodothyronine (fT3), and free thyroxine (fT4), were performed in the study cohort within 3 months before and 3 months after COVID-19. The three-month pre- and post-infection window was selected to capture thyroid function measurements obtained close to the COVID-19 episode, while minimizing the influence of unrelated long-term changes. The study protocol was approved by the appropriate institutional ethics committee and conducted in accordance with the Declaration of Helsinki. COVID-19 status was confirmed via a positive SARS-CoV-2 antigen test. Pre- and post-infection thyroid function tests were performed in the same laboratory using the same analytical methods and reference ranges. Patients without available thyroid function measurements within the predefined study periods were excluded from the analysis; no imputation of missing data was performed. SAT was diagnosed based on clinical symptoms, laboratory findings, and characteristic thyroid ultrasound findings. The pre-COVID-19 thyroid function test results were obtained retrospectively from the patients’ medical records. These tests had been performed as part of routine primary care or clinical evaluation prior to SARS-CoV-2 infection, rather than specifically for the purposes of the present study. The specific clinical indication for thyroid function testing was not systematically recorded and, therefore, could not be determined for each patient.

### Statistical Analysis

Statistical analysis was performed using a paired Student’s *t*-test for dependent samples to compare thyroid function parameters before and after COVID-19. Prior to analysis, the distributions of the analyzed variables were visually inspected, and summary distribution characteristics were reviewed. The results were interpreted based on *p*-values, with a *p*-value < 0.05 considered statistically significant. All statistical analyses were performed using IBM SPSS Statistics version 20 (IBM Corp., Armonk, NY, USA), while Microsoft Excel for Mac (Microsoft 365; Microsoft Corporation, Redmond, WA, USA) was used for data management and preparation of figures.

## 3. Results

The final analysis included 373 patients without previously diagnosed thyroid disease. Women comprised the majority of the study population (*n* = 267), and the mean age of participants was 63.8 ± 16.7 years (median age: 66 years).

Table 1 presents the mean values, standard deviations, and median concentrations of selected thyroid parameters measured before and after COVID-19. A statistically significant reduction in TSH concentration was observed after COVID-19, when compared with pre-infection values (*p* < 0.05); in contrast, no statistically significant differences were observed for fT3 or fT4 concentrations.

Although a statistically significant reduction in the TSH level was observed in the overall study population, no statistically significant differences were found in gender- or age-adjusted subgroup analyses. No statistically significant changes were observed in fT3 or fT4 concentrations, and no correlations were identified in gender- or age-adjusted analyses.

**Figure 1:**
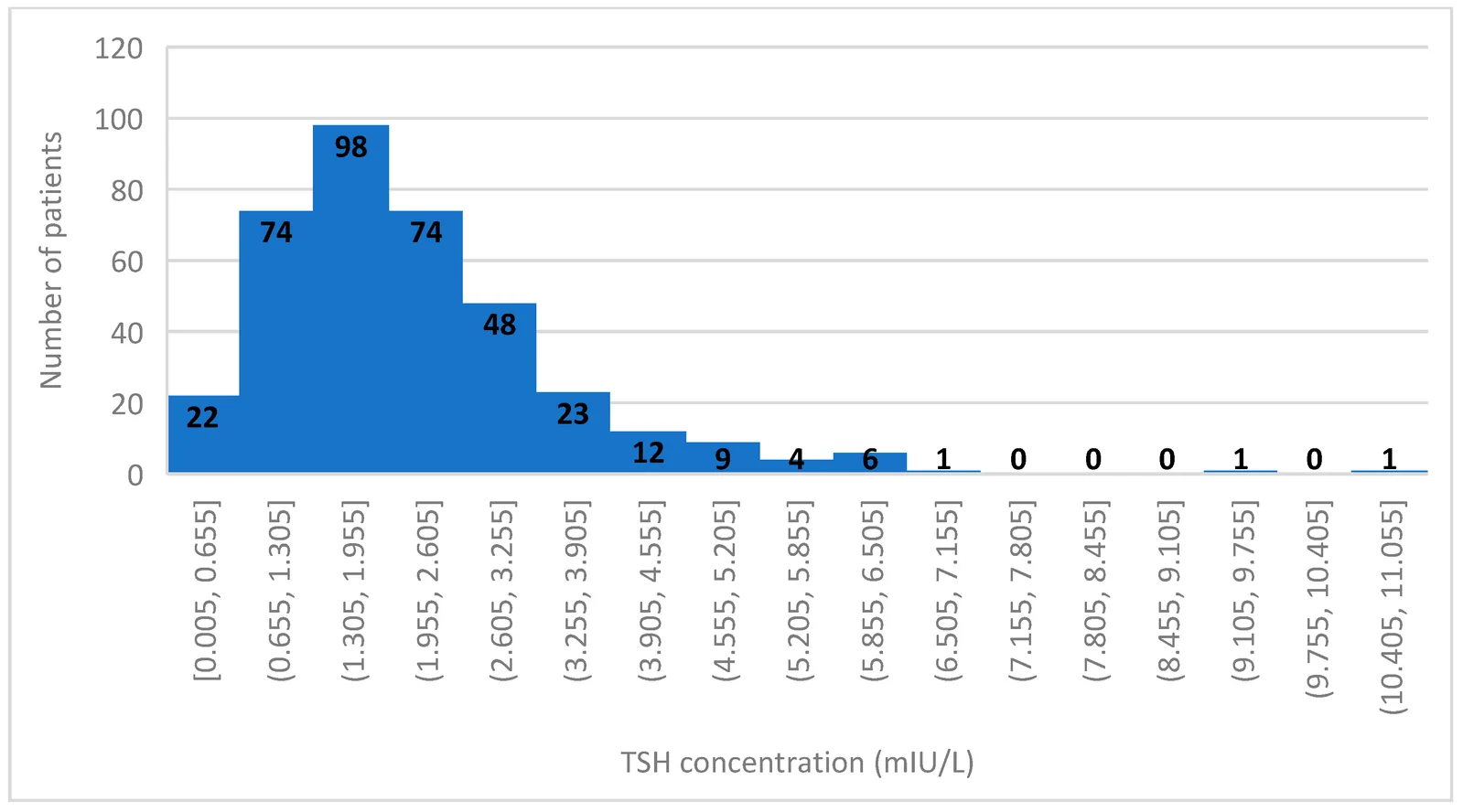
Distribution of thyroid-stimulating hormone (TSH) concentrations measured before COVID-19.

Figure 1 presents the distribution of TSH concentrations measured before COVID-19, from which it can be seen that the majority of patients (*n* = 98) had TSH values within the range of 1.305–2.605 mIU/L.

**Figure 2:**
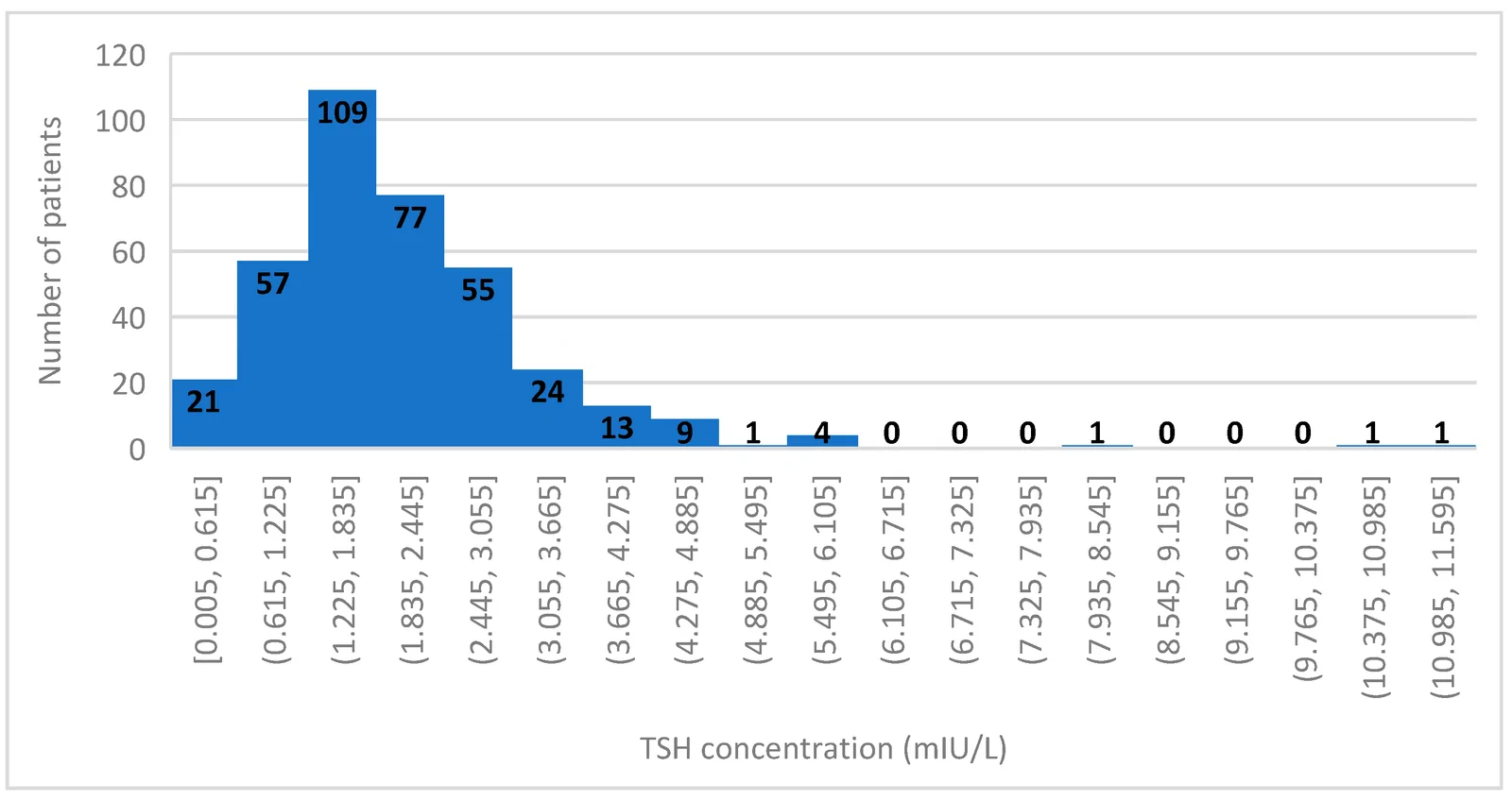
Distribution of TSH levels after COVID-19.

Figure 2 presents the distribution of TSH levels measured after COVID-19.

After COVID-19, most patients (*n* = 109) had TSH concentrations within the range of 1.22–1.83 mIU/L. Low TSH levels (i.e., ranging from 0.005 to 0.615 mIU/L) were observed in 21 patients after COVID-19; for comparison, 22 patients had TSH concentrations within the same range prior to infection.

**Figure 3:**
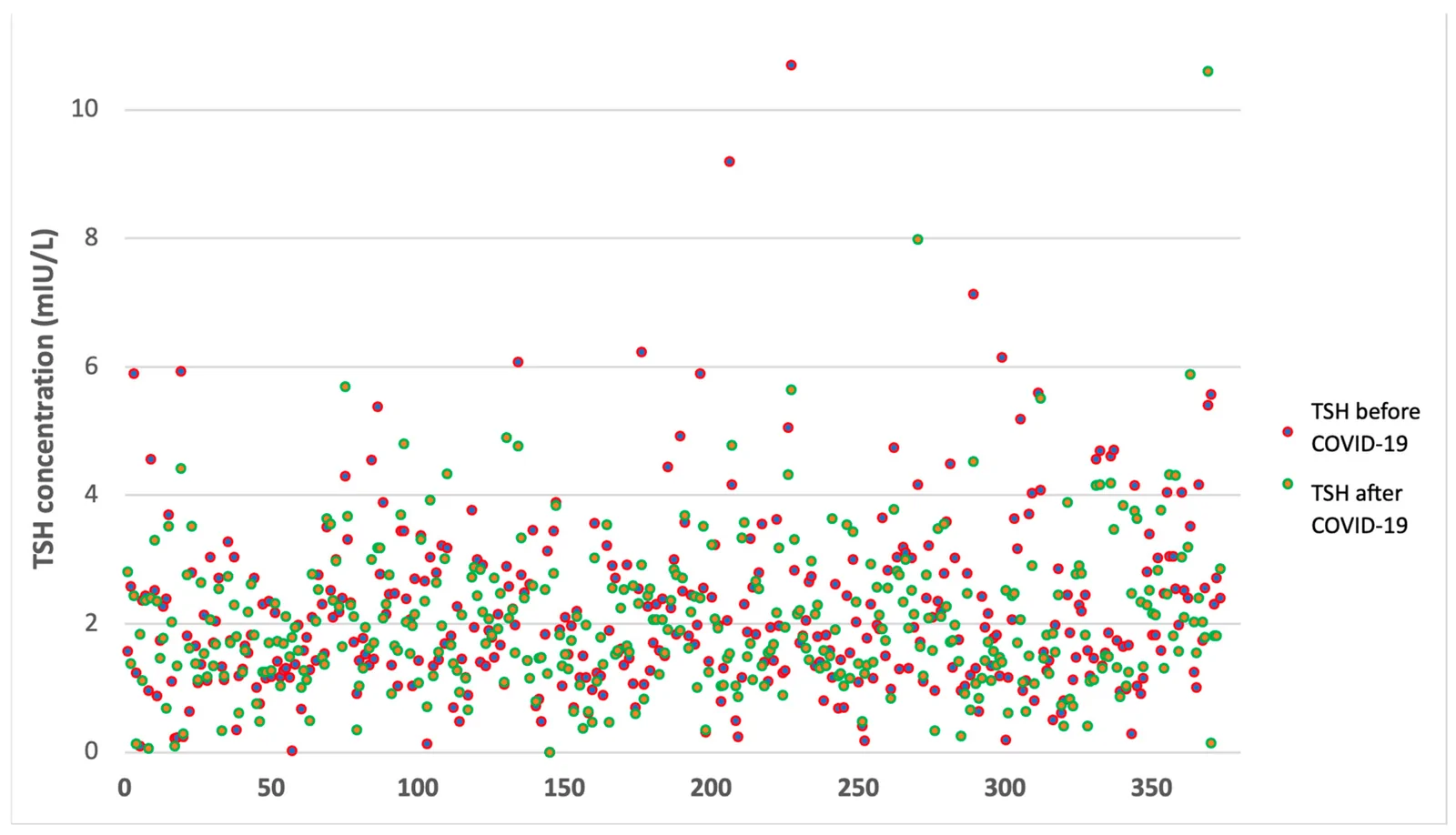
Individual changes in thyroid-stimulating hormone (TSH) concentrations in study participants, measured before and after COVID-19.

Figure 3 presents the individual changes in TSH levels in study patients, measured before and after COVID-19.

The mean TSH concentration after COVID-19 was significantly lower when compared with the pre-infection mean (*p* < 0.05).

## 4. Discussion

SAT is usually a self-limiting inflammatory disease, the activation of which is frequently associated with viral infections and vaccinations [4]. Neck pain radiating to the jaw or the ear and general symptoms of fever, fatigue, and muscle aches are the most common reasons for a patient to report to a primary care center due to SAT [30,31,32]. The general clinical presentation of inflammation, as described above, is also associated with the presence of elevated inflammatory markers such as C-reactive protein (CRP) and the erythrocyte sedimentation rate (ESR). Subsequent laboratory tests for thyroid function, as routinely conducted in patients reporting these complaints, frequently reveal early stages of hyperthyroidism along with elevated fT4 and suppressed TSH levels; meanwhile, in patients with hypothyroidism, TSH levels increase while fT4 levels are suppressed [10,13,15]. In the course of SAT, the antithyroid antibody screening results may remain unremarkable, although transient elevation in the levels of anti-TPO and anti-Tg antibodies has been reported. Such findings have been proposed to reflect immune activation associated with COVID-19; however, the underlying mechanisms remain uncertain.

In the context of SAT, ultrasound (US) examinations of the thyroid show an enlarged, hypoechoic gland with heterogeneous structure and hypoperfusion [32]. The treatment of SAT is centered around the administration of non-steroidal anti-inflammatory drugs (NSAIDs) as first-line medications (especially in mild cases), due to their efficacy in eliminating pain and shortening the duration of discomfort [33,34]. The treatment of patients with more severe SAT who fail to respond to NSAID therapy proceeds with the administration of glucocorticosteroids (GCSs; it should be noted, however, that GCSs do not prevent hypothyroidism) [18,35]. Beta-blockers may also be used to control thyrotoxicosis symptoms (e.g., tremor, palpitations) [16,31,36]. In chronic hypothyroidism (which has been estimated to occur in 10–15% of patients), levothyroxine replacement therapy may be necessary [34,37]. The mechanism of SAT activation due to triggers such as viral infections and prophylactic interventions (vaccinations) appears to remain valid in the case of new pathogens, including SARS-CoV-2. Although the development of SAT is a rare complication following SARS-CoV-2 vaccination, its troublesome symptoms make it an undesirable condition that requires further research and analysis. It is worth noting that most of the reported cases have occurred in women [4,38,39].

Activation of SAT has been observed with various types of vaccines, including mRNA, viral vector, and inactivated vaccines [24,25]. Although SAT symptoms are usually observed within 10 days of vaccination, their onset can take place from as early as 2 days up to as late as several weeks after vaccination. The general symptoms are characteristic of SAT, as are the laboratory findings (inflammatory and thyroid markers), although asymptomatic or euthyroid states and thyrotoxic periodic paralysis (TPP, most frequently observed in males of Asian descent) have been reported [40]. In terms of the laboratory results, the hallmarks of SAT usually include thyrotoxicosis characterized by low TSH levels, elevated fT3 and fT4 levels, and elevated levels of inflammatory markers such as CRP and the ESR [24,25]. In this study, a statistically significant reduction in the TSH level was observed in the pre- vs. post-infection comparison, while no statistically significant changes were observed in the fT3 and fT4 levels. Recent evidence suggests that there is considerable variability in COVID-19-related thyroid function alterations, which are potentially influenced by disease severity, the timing of thyroid function assessment, and the clinical characteristics of the studied population [41,42]. Despite reaching statistical significance, the decrease in the mean TSH level was relatively small (from 2.39 to 2.17 mIU/L), and no corresponding changes were observed in fT3 or fT4 concentrations. Furthermore, the observed decrease in the mean TSH concentration, from 2.39 to 2.17 mIU/L, was accompanied by a slight increase in the median TSH concentration, from 1.85 to 1.92 mIU/L. The difference in the direction of change in these measures of central tendency suggests the possibility of an asymmetric distribution of TSH values, justifying caution in the clinical interpretation of the observed difference. Consequently, the clinical relevance of the observed change is difficult to determine. The isolated change in the TSH level may reflect transient alterations in the hypothalamic–pituitary–thyroid axis following infection, although biological variability, differences in measurement timing, and regression to the mean may also have contributed. Due to the observational study design and the lack of a non-COVID-19 control group, it is not possible to establish a causal relationship between SARS-CoV-2 infection and the observed changes in thyroid function. No TSH suppression was observed in the laboratory findings. The stability of the fT3 and fT4 concentrations suggests that the observed thyroid alterations were generally mild and did not result in overt thyroid dysfunction in most patients. Another possible explanation for the observed thyroid function changes may be non-thyroidal illness syndrome (NTIS), which has been reported in patients with COVID-19. However, we consider this explanation less likely due to the absence of significant changes in fT3 and fT4 concentrations. Post-COVID-19 immune dysregulation and inflammatory responses affecting the hypothalamic–pituitary–thyroid axis may represent one possible mechanism underlying the observed thyroid function alterations; however, this hypothesis cannot be directly evaluated in the present study, as thyroid autoantibodies, inflammatory cytokines, and other markers of autoimmune activity were not assessed. From a primary care perspective, these findings do not support routine endocrine intervention in asymptomatic patients, but may justify thyroid function assessment in individuals presenting with persistent symptoms suggestive of thyroid dysfunction following COVID-19. As the clinical symptoms of SAT may not be typical in these individuals, ultrasound examination of the thyroid gland can be helpful in establishing a correct diagnosis. Parenchymal lesions within the thyroid include bilateral or unilateral, localized or multifocal hypoechoic areas. In four studies published on this topic, symptoms of thyrotoxicosis were reported in three cases, with the exception of one case of SAT associated with COVID-19 (all patients were female, aged 18 to 69 years). Subacute thyroiditis developed between days 5 and 30 after the COVID-19 diagnosis, and the recovery period ranged from 7 to 30 days [15,20,21]. In a study by Resulti et al., 5 patients aged between 21 and 45 presented with no signs of thyrotoxicosis. The average recovery time was 25 days [24]. The three typical clinical manifestations of SAT are hyperthyroidism, transient hypothyroidism, and euthyroidism. The euthyroid state is usually reached within 3 months (I), and persistent hypothyroidism occurs in about 10–36.5% of patients [17,18,20].

### 4.1. Limitations

Despite its strengths, this study has several limitations that should be acknowledged. First, its retrospective single-center design may limit the generalizability of the reported findings. Second, the study did not include a control group of individuals without COVID-19, which precludes causal inference regarding the observed thyroid function changes. Moreover, temporal variation in thyroid function and regression to the mean cannot be excluded as alternative explanations for the observed reduction in TSH levels. Third, inclusion was restricted to patients with available thyroid function measurements before and after COVID-19, which may have introduced selection bias. Furthermore, information on several potentially relevant confounding factors, including body mass index, smoking status, comorbidities, medication use, COVID-19 severity, vaccination history, and therapeutic interventions, was not consistently available and therefore could not be incorporated into the analysis. In addition, no multivariable regression analysis was performed to adjust for potential confounders. No effect size estimates or 95% confidence intervals were presented, and a lack of access to individual data precluded additional nonparametric analyses for paired data. The study also lacked long-term follow-up, preventing assessment of the persistence or resolution of the observed hormonal changes over time. Finally, although a statistically significant reduction in TSH concentrations was observed, the magnitude of this change was modest, and its clinical significance remains uncertain. Therefore, the findings should be interpreted as descriptive and hypothesis-generating, rather than confirmatory.

### 4.2. Future Research Perspectives

Future prospective multicenter studies with longer follow-up periods are needed to better characterize the long-term effects of COVID-19 on thyroid function. In addition, researchers should further explore factors that may influence post-COVID-19 thyroid function changes, including patient characteristics, disease course, and therapeutic interventions.

## 5. Conclusions

This study provides additional insight into the changes in thyroid function occurring following COVID-19 in a real-world primary care population. A modest decrease in the TSH level was observed after COVID-19, while the fT3 and fT4 concentrations remained largely unchanged. The clinical significance of the observed changes remains uncertain. Given the retrospective study design, absence of a non-COVID-19 control group, and lack of adjustment for potential confounding factors, these findings should be interpreted with caution and do not allow causal conclusions to be drawn. As primary care is often the first point of contact for patients presenting with persistent or new symptoms following COVID-19, thyroid function assessment may be considered when clinically indicated. Further prospective, controlled studies are needed to determine the clinical significance of thyroid function changes following COVID-19.

## Figures and Tables

**Table 1:** Concentrations of selected thyroid function parameters before and after COVID-19.

Parameter	Before COVID-19	After COVID-19
TSH, mean	2.39	2.17
TSH, SD	4.11	1.87
TSH, median	1.85	1.92
fT3, mean	2.93	2.98
fT3, SD	0.50	1.02
fT3, median	2.91	2.93
fT4, mean	1.31	1.30
fT4, SD	0.28	0.27
fT4, median	1.29	1.27

TSH, thyroid-stimulating hormone; fT3, free triiodothyronine; fT4, free thyroxine. Values are presented as mean, standard deviation (SD), and median. Units: TSH (mIU/L), fT3 (pg/mL), fT4 (ng/dL).

## Data Availability

The datasets generated and/or analyzed during the current study are not publicly available due to data protection and privacy regulations, but are available from the corresponding author on reasonable request.

## Abbreviations

COVID-19	Coronavirus disease 2019
CRP	C-reactive protein
ESR	Erythrocyte sedimentation rate
fT3	Free triiodothyronine
fT4	Free thyroxine
GCSs	Glucocorticosteroids
NSAIDs	Non-steroidal anti-inflammatory drugs
NTIS	Non-thyroidal illness syndrome
SAT	Subacute thyroiditis
SARS-CoV-2	Severe acute respiratory syndrome coronavirus 2
TPP	Thyrotoxic periodic paralysis
TSH	Thyroid-stimulating hormone
US	Ultrasound
